# Evidence for prodromal changes in neuronal excitability and neuroinflammation in the hippocampus in young alpha-synuclein (A30P) transgenic mice

**DOI:** 10.3389/frdem.2024.1404841

**Published:** 2024-06-17

**Authors:** Ibtisam Al-Musawi, Bethany H. Dennis, Gavin J. Clowry, Fiona E. N. LeBeau

**Affiliations:** Biosciences Institute and Centre for Transformative Neuroscience, Faculty of Medical Sciences, Newcastle University, Newcastle upon Tyne, United Kingdom

**Keywords:** alpha-synuclein, neuroinflammation, hippocampus, glial cells, hyperexcitability

## Abstract

**Introduction:**

Neuronal hyperexcitability and neuroinflammation are thought to occur at early stages in a range of neurodegenerative diseases. Neuroinflammation, notably activation of microglia, has been identified as a potential prodromal marker of dementia with Lewy bodies (DLB). Using a transgenic mouse model of DLB that over-expresses human mutant (A30P) alpha-synuclein (hα-syn) we have investigated whether early neuroinflammation is evident in the hippocampus in young pre-symptomatic animals.

**Methods:**

Previous studies have shown early hyperexcitability in the hippocampal CA3 region in male A30P mice at 2–4 months of age, therefore, in the current study we have immunostained this region for markers of neuronal activity (c-Fos), reactive astrocytes (glial fibrillary acidic protein, GFAP), microglia (ionizing calcium binding adapter protein 1, Iba-1) and reactive microglia (inducible nitric oxide synthase, iNOS).

**Results:**

We found an interesting biphasic change in the expression of c-Fos in A30P mice with high expression at 1 month, consistent with early onset of hyperexcitability, but lower expression from 2–4 months in male A30P mice compared to wild-type (WT) controls, possibly indicating chronic hyperexcitability. Neuroinflammation was indicated by significant increases in the % area of GFAP and the number of Iba-1+ cells that expressed iNOS immunoreactivity in the CA3 region in 2–4 months A30P male mice compared to WT controls. A similar increase in % area of GFAP was observed in female A30P mice, however, the Iba-1 count was not different between female WT and A30P mice. In WT mice aged 2–4 months only 4.6% of Iba-1+ cells co-expressed iNOS. In contrast, in age matched A30P mice 87% of cells co-expressed Iba-1 and iNOS. Although there was no difference in GFAP immunoreactivity at 1 month, Iba-1/iNOS co-expression was also increased in a cohort of 1 month old A30P mice.

**Discussion:**

Abnormal hα-syn expression in A30P mice caused early changes in network excitability, as indicated by c-Fos expression, and neuroinflammation which might contribute to disease progression.

## Introduction

Lewy body dementia (LBD), which includes dementia with Lewy bodies (DLB), Parkinson's disease (PD), and Parkinson's disease dementia (PDD) is the second most common form of dementia (McKeith et al., 2020) after Alzheimer's disease (AD). LBDs are a group of progressive neurodegenerative diseases, classed as alpha-synucleinopathies, that are caused by the abnormal accumulation of the pre-synaptic protein alpha-synuclein (α-syn) into toxic oligomers and fibrils that form the Lewy bodies (Spillantini et al., 1998). These toxic forms of clustered α-syn then lead to synaptic dysfunction, network oscillation abnormalities, and ultimately cell death (Saramowicz et al., 2023; Nordengen and Morland, 2024). Chronic neuroinflammation is evident in PD brains where microglia activation is seen in areas with high levels of α-syn aggregates (Iba et al., 2020, 2023). Although the exact mechanisms by which α-syn causes neurodegeneration are still uncertain, there is evidence for a link between α-syn pathology, neuroinflammation and changes in cortical excitation/inhibition and cognitive function (Deyell et al., 2023).

Microglia are phagocytic cells that are known to show an aberrant, activated phenotype and produce pro-inflammatory cytokines in neurodegenerative diseases including the alpha-synucleinopathies (Mavroeidi and Xilouri, 2021; Deyell et al., 2023). Aberrant α-syn activates microglia leading to increased phagocytic activity aimed at clearing toxic material, but long-term activation and chronic inflammation ultimately leads to cell death and neurodegeneration (Cardinale et al., 2021; Chavarria et al., 2022). Activated microglia also release reactive oxygen/nitrogen species such as nitric oxide (NO) produced via inducible nitric oxide synthase (iNOS). Elevated iNOS in both neurons and microglia has been seen in post-mortem LBD patient's brains and mouse models of PD (Stykel and Ryan, 2022; Loveland et al., 2023). In DLB microglia activation has been proposed to occur early in the prodromal phase of the disease progression. Positron emission tomography (PET) imaging, using a ligand that binds to a translocator protein receptor (TSPO), expressed on the outer mitochondrial membrane of activated microglia, has shown increased reactive microglia in early prodromal DLB (Iannaccone et al., 2013; Surendranathan et al., 2015). Data from rodent studies have shown that mouse models using intracranial α-syn preformed fibril injection, or over-expression of wild type or mutant human α-syn, all exhibit activation of microglia but with model, brain region or age-dependent differences reported (Gomez-Isla et al., 2003; Watson et al., 2012; Gentzel et al., 2021; Torres et al., 2021).

Astrocytes play key roles in a wide range of neuronal homeostatic functions including regulating synaptic transmission and metabolic support (Ben Haim and Rowitch, 2017) but are also activated by α-syn pathology, and activated astrocytes contribute to the neuroinflammatory response in neurodegenerative diseases (Liddelow and Barres, 2017; Di Marco Vieira et al., 2020; Mavroeidi and Xilouri, 2021; Yi et al., 2022). In response to an immune signal, astrocytes switch from a resting, surveillance mode, to a reactive state and express glial fibrillary acid protein (GFAP). Increased numbers of GFAP expressing cells have been seen in post-mortem human brain tissue from patients with DLB (Iseki et al., 2000; Togo et al., 2001).

Activation of both astrocytes and microglia, and the resulting increase in cytokine release, can lead to network hyperexcitability (Vezzani and Viviani, 2015), which could then contribute to disease progression. There is a clear link between neuroinflammation and network hyperexcitability in conditions such as epilepsy (Li et al., 2023; Yu et al., 2023). More recently neuronal hyperexcitability has also been linked to early pathophysiological changes in neurodegenerative diseases, most extensively studied in relation to AD, but also DLB (Vicente et al., 2024). AD is associated with excitatory/inhibitory network imbalances and an increased risk of epilepsy (Vossel et al., 2016; Beagle et al., 2017). Although only a small proportion of patients with AD have overt seizures, many more patients are thought to have subclinical seizures (Lam et al., 2017). More recently studies have suggested increased excitation also occurs in DLB. Patients with DLB have an increased risk of seizures or myoclonus, a form of cortical hyperexcitability (Beagle et al., 2017; Marawar et al., 2020), and again seizures can be subclinical (Musaeus et al., 2023) and thus go undetected.

Cortical hyperexcitability has long been known to occur in murine models of AD (Palop et al., 2007). Previously in the same 2–4 months old human mutant α-syn expressing A30P mice (Kahle et al., 2000) used in the present study we reported spontaneous interictal discharges *in vitro* in the hippocampus following application of kainate to induce gamma frequency oscillations (Tweedy et al., 2021). This was never seen in slices from young wild type mice. In addition, we showed that the threshold for interictal activity in response to application of the GABA_A_ receptor antagonist gabazine (SR95531) was significantly lower in the young A30P mice. *In vivo* under urethane anesthesia we found a faster sleep-related slow oscillation frequency, also consistent with increased excitability (Stylianou et al., 2020). Other groups have also shown interictal and seizure-like activity in transgenic mice expressing human wild type alpha-synuclein (Morris et al., 2015), or the A53T mutation (Peters et al., 2020), which all suggests hyperexcitability is also a key feature of mice with alpha-synuclein pathology.

In the current study we aimed to investigate whether early neuronal hyperexcitability, reported in the transgenic hα-syn mouse lines, was linked to early neuroinflammatory changes. Using immunofluorescence (IF) our results showed an early up-regulation of c-Fos, an indirect marker of neuronal activity, in the hippocampus of A30P juvenile mice compared to WT (1 month old), that was followed by down-regulation of c-Fos in the young adult A30P mice (2–4 months), suggesting chronic network hyperexcitability (Kawashima et al., 2014). The c-Fos changes were accompanied by an increase in both reactive GFAP+ astrocytes and in the proportion of microglia that co-expressed iNOS in A30P male mice compared to WT aged 2–4 months. Similar evidence for reactive microglia was also seen in A30P mice as young as 1 month. The data clearly demonstrated that neuroinflammation was an early occurrence arising from the abnormal expression of mutant human α-synuclein in A30P mice, and our findings are consistent with the prodromal neuroinflammatory changes seen in patients with DLB.

## Methods

### Transgenic mice and animal housing

Both male and female α-syn transgenic mice expressing human mutant α-syn (A30P) under the control of the Thy-1 promoter (Kahle et al., 2000) were used in this study. The A30P mice were bred in house from homozygous breeding pairs originally supplied by Dr. P Kahle, University of Tubingen. A homozygous A30P line was subsequently re-generated using control C57BL/6 mice from Charles River (Tranent, UK). Heterozygous offspring were genotyped (Transnetyx, Cordova, TN, USA) and new homozygous lines of both control and A30P mice were established with the F1 offspring further genotyped. Control mice were age matched C57BL/6 either bred in house or purchased from Charles River Laboratories (Harlow, UK). Animals were housed according to ARRIVE guidelines and were maintained on a 12-h dark/light cycle with lights on at 7.00 a.m.

### Animal perfusion and immunofluorescence

All procedures were in accordance with the UK Animals (Scientific Procedures) Act 1986 and European Union directive 2010/63EU. All mice were anesthetized with inhaled isoflurane prior to intramuscular injection of ketamine (≥100 mg kg^−1^) and xylazine (≥10 mg kg^−1^). When all responses to noxious stimuli, such as pedal withdrawal reflex, had terminated the animals were intracardially perfused with ~25–30 ml 0.9% NaCl solution followed by ~50 mls of 4% buffered paraformaldehyde (PFA).

Brains were either stored in PFA at 4°C or, if kept for longer than a week, in cryopreservant (glycerol and ethylene glycol in deionised water and 0.3 M PBS) at −20°C and then transferred to 30% sucrose solution in phosphate buffered saline (PBS) pH 7.4 overnight prior to sectioning. Horizontal sections (35 μm) were cut on a freezing microtome and collected free floating in 0.1 M PBS. Sections were incubated in 3% normal donkey serum (NDS), 0.3% Triton x-100 in PBS for 3 h on a rotary shaker at 4°C, before being incubated overnight in primary antibody diluted in 3% NDS, 0.3% Triton X-100 in PBS (for dilutions see Table 1). Sections were than washed for 3 × 10 min in PBS + 0.3% Triton (PBST) and then appropriate secondary antibodies diluted in PBST were applied (see Table 2) and the sections incubated at room temperature for 3 h. Sections were protected from light to avoid the bleaching of the secondary antibodies and then underwent three 10-min washes in PBS and were transferred onto gelatine-coated glass slides. Once the transferred sections had dried, they were mounted in Fluoromount-G mounting media with DAPI^TM^ (4', 6-diamide-2-phenylindole; Abcam) and coverslipped. The slides were left at room temperature to dry fully and then subsequently stored at 4°C prior to imaging.

**Table 1:** Primary antibodies used.

**Antibody to**	**Definition**	**Host**	**Supplier**	**RRID**	**Dilution (v:v)**
hα-syn	Human alpha-synuclein	Rat	Enzo Life Sciences, Exeter, UK	AB 2050691	1:250
GFAP	Glial fibrillary acidic protein	Rabbit	Dako, Ely, UK	AB 10013382	1:2,000
Iba-1	Ionized calcium-binding adapter molecule 1	Goat	Abcam, Cambridge, UK	AB 222402	1:2,000
c-Fos	Immediate early gene (IEG)	Rabbit	Abcam, Cambridge, UK	AB 2891049	1:500
iNOS	Inducible nitric oxide synthase	Rabbit	Abcam, Cambridge, UK	AB 3083470	1:200

**Table 2:** Secondary antibodies used.

**Secondary antibody**	**Manufacturer**	**Dilution (v:v)**
Donkey anti-rabbit IgG, Alexa Fluor 594	ThermoFisher Scientific	1:1,000
Donkey anti-goat IgG, Alexa Fluor 488	Abcam	1:1,000
Donkey anti-rat IgG, Alexa Fluor 594	Invitrogen	1:1,000

### IF imaging and analysis

**Figure 1:**
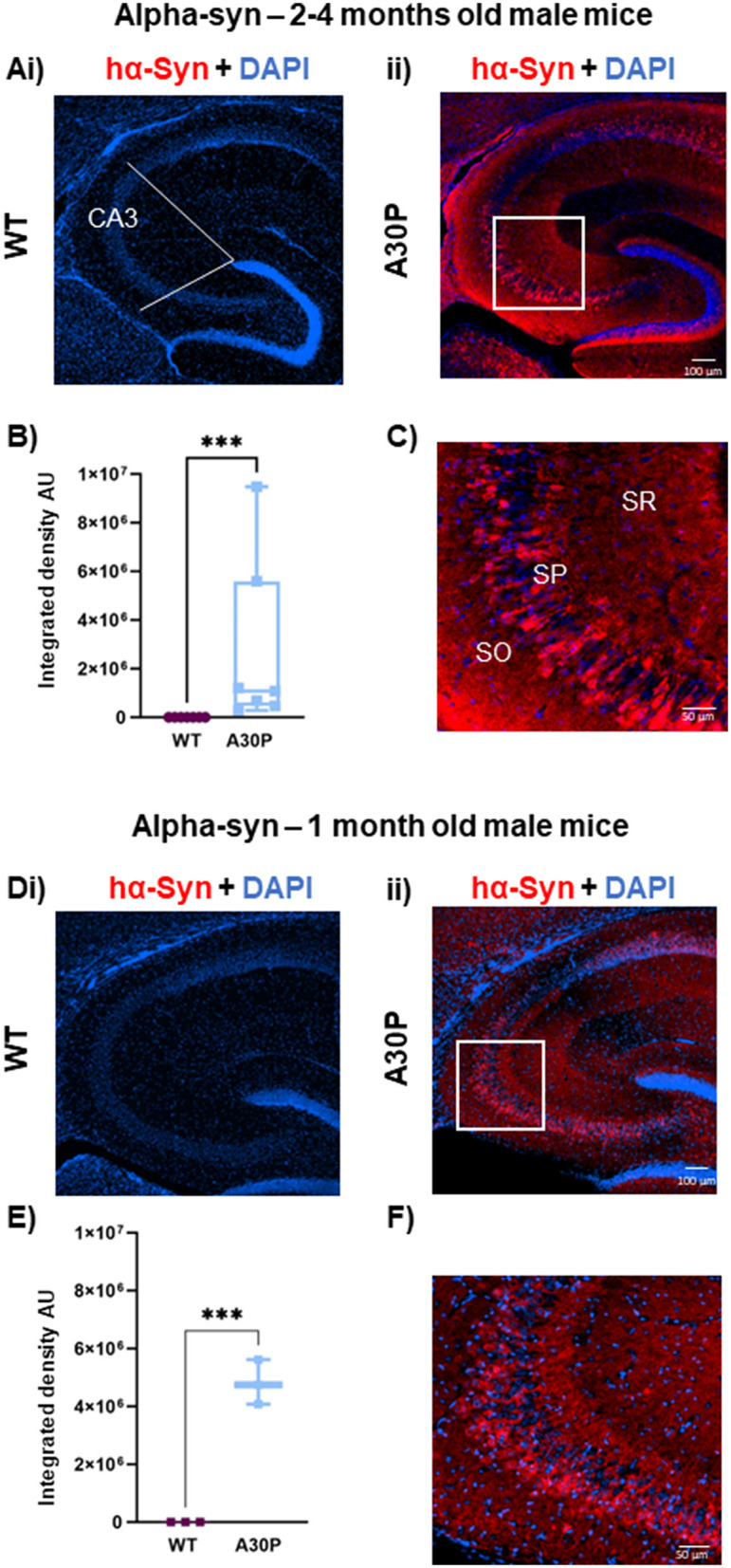
Mutant hα-syn expression predominates in deep layer CA3 pyramidal cells: **(Ai)** IF staining for hα-syn (red) with DAPI counterstain (blue) in 2–4 months old male mice shows no staining in **(Ai)** WT but clear labeling in young 2–4 months old **(Aii)** A30P mice. White lines indicate the ROI in CA3. **(B)** Quantification of the integrated density showed a significantly (*p* < 0.001) greater integrated density (ID) of α-syn in CA3 in A30P mice. **(C)** In the expanded view (from box in **Aii**) hα-syn labeling can be seen predominantly in the deep *stratum pyramidale* (SP) cell layer. Similar results were seen in **(Di)** WT vs. **(Dii)** A30P mice aged 1 month. **(E)** Shows a significant increase in hα-syn integrated density in A30P compared to WT mice (*p* < 0.001). **(F)** and hα-syn expression again appeared stronger in the deep pyramidal cell layers in CA3. ***p < 0.001.

Sections were imaged using an upright Nikon Eclipse Ni-E fluorescent microscope equipped with an Andor Zyla 5.2 camera and the Nikon Elements software. Sections were imaged with a 10x or 20x objective and throughout image capture, for each data set, all parameters including lamp intensity and calibration were kept the same. Fiji software (https://imagej.net/software/fiji) was used for analysis. Images were converted to a binary image using a threshold value, fluorescence was enhanced, and the noise was reduced. Brightness and contrast were then kept constant for all analysis within a data set. Because our previous studies (Tweedy et al., 2021) have shown increased network excitability in the CA3 region of the hippocampus we focused our analysis on this region. To quantify changes in CA3 a region of interest (ROI) was measured from the blade of the dentate gyrus to the edge of CA2 and encompassed all layers of the CA3 area (Figure 1A). For the laminar distribution analysis, three ROIs were measured consisting of *stratum oriens* (SO), *stratum pyramidale* (SP) and *stratum lucidum*/*radiatum* (SR). Values for integrated density of fluorescence, cell count and percentage (%) area occupied by immunofluorescent structures in the ROI (CA3) were obtained. For all analysis sections were imaged in two dimensions and ROI data (cell counts and % area) were reported per mm^2^. For the co-labeling of Iba-1 and iNOS we used compressed Z-stacks of the ROI with a depth of 3 × 10 μm but cell counts were made on the compressed image and thus also reported as per mm^2^. For quantitative analysis at least three sections per mouse were used and group sizes were at least three mice but usually six to eight mice were used. Data were reported as *n*/*N* = section/mouse numbers. Statistics were performed on average values for each mouse.

### Statistical analysis

Data were statistically analyzed and plotted using Prism 9.0 (GraphPad Software, USA). Data were tested for normality (Shapiro-Wilk test). Most of the data were found to be not normally distributed, therefore, for consistency most data was treated as non-parametric and plotted as box plots showing median and interquartile range (IQR). To compare two independent samples a non-parametric Mann-Whitney *U*-test was performed. To compare dependent variables (laminar distributions) a Friedman's test was performed on non-parametric data followed by a Dunn's multiple comparison test. Significance values were reported as ^*^p < 0.05, ^**^p < 0.01, ^***^p < 0.001.

## Results

### hα-syn expression in deep vs. superficial pyramidal cell layers

We have previously shown increased network excitability in the CA3 regions of the hippocampus in A30P mice at 2–4 months (Tweedy et al., 2021), several months prior to the onset of cognitive and motor dysfunction which occur at ~12 and ~14 months, respectively (Kahle et al., 2000; Freichel et al., 2007). To determine a possible role for neuroinflammation in the early network excitability changes, in the current study we have used mice aged either 1 month or 2–4 months, and first confirmed the expression of human α-syn in these two age groups (Figure 1). As expected, in adult mice aged 2–4 months no hα-syn immunoreactivity was evident in the WT mice confirming the specificity of the antibody for hα-syn (Figure 1Ai), but immunoreactivity was clearly seen across the whole hippocampus in all A30P mice (Figure 1Aii). In the A30P line hα-syn expression is under the control of the Thy-1 promoter, which is predominantly, but not exclusively, active in pyramidal cells (Sugino et al., 2006), and we saw clear staining of a proportion of cell bodies in the pyramidal cell layer and staining throughout the hippocampal neuropil that might reflect synaptic terminals (Figure 1Aii). There was a significant increase in hα-syn integrated density in A30P mice (Figure 1B), although the median integrated density did vary across animals (1,081,426, IQR 471,975–5,585,435; *n*/*N* = 54 sections/seven mice vs. WT median 308, IQR 270–333; *n*/*N* = 41 sections/seven mice; *p* < 0.001, Mann Whitney test). Interestingly, we observed that hα-syn expression appeared to be greater in neurons in the deep layers (Slomianka et al., 2011) of the CA3 pyramidal cell layer (Figure 1C).

Previous studies have shown hα-syn is expressed in A30P mice from 1 month of age (Kahle et al., 2000), and this was confirmed in the current study. Again, there was no immunoreactivity in WT mice (Figure 1Di), but hα-syn was evident in the A30P mice (Figure 1Dii). There was a significant increase (Figure 1E) in hα-syn in A30P mice (mean integrated density 4,810,731 ± 444,746, *n*/*N* = 10 sections/three mice vs. WT mean integrated density 248 ± 13.48; *n*/*N* = 10 sections/three mice; *p* < 0.001, Unpaired *t*-test). Again, the expanded view shows a preferential distribution of hα-syn to neurons situated in the deep pyramidal cell layers (Figure 1F).

### Age-dependent biphasic changes in c-Fos expression in A30P mice

**Figure 2:**
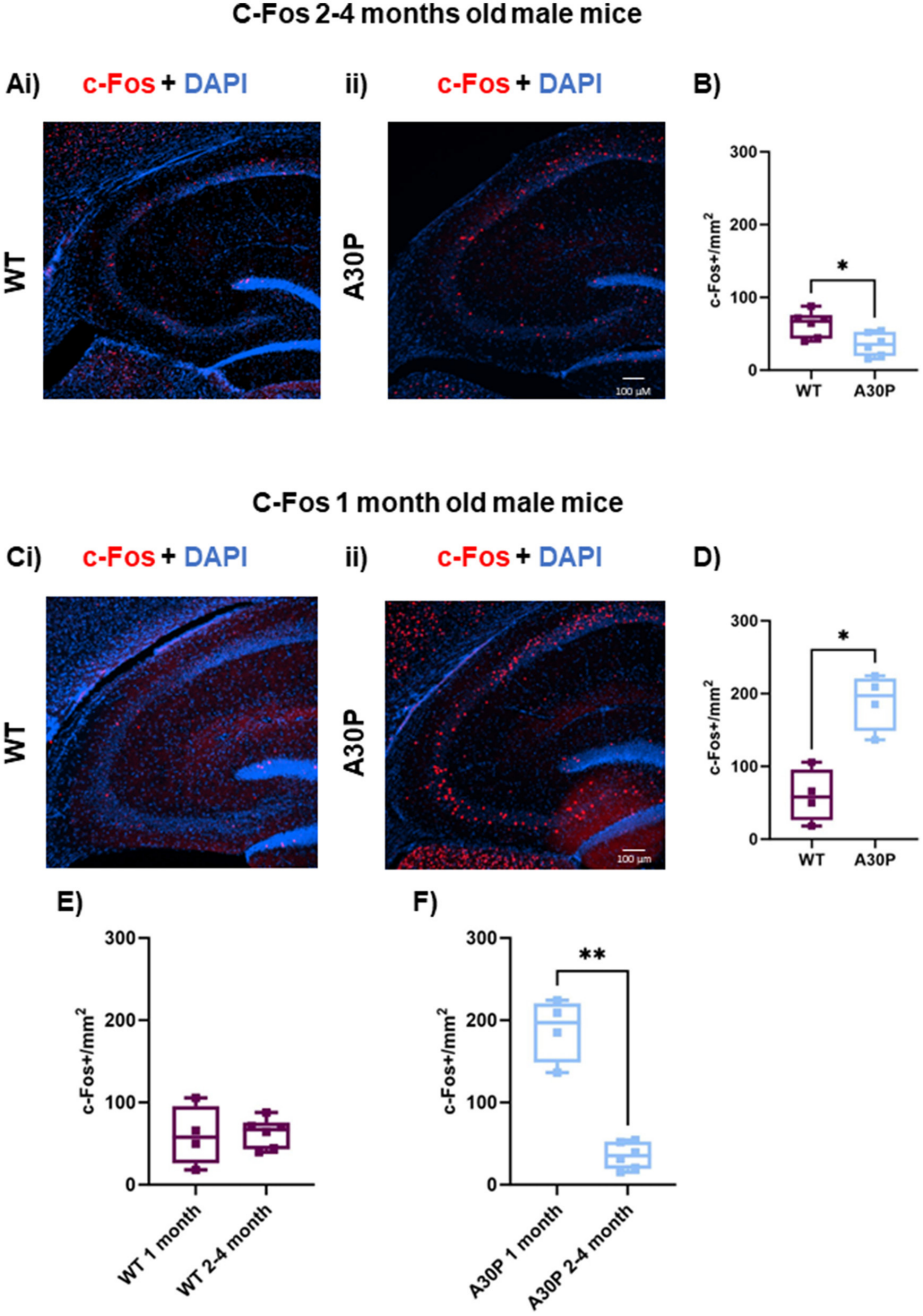
Age-dependent biphasic regulation of c-Fos expression in 2–4 and 1 month old A30P mice. **(Ai)** IF staining for c-Fos (red) with DAPI counterstain (blue) in WT male mice 2–4 months old shows a few cells expressing c-Fos throughout the CA3 region of the hippocampus. **(Aii)** In A30P mice there is a decrease in c-Fos compared to WT. **(B)** Quantification of c-Fos cell count showed a significant (*p* < 0.05) reduction in c-Fos staining in the 2–4 months old A30P mice. At 1 month old mice **(Ci)** in the WT c-Fos counts were similar to those seen in the 2–4 months old WT mice, but in the A30P mice **(Cii)** there were markedly more c-Fos labeled cells across the hippocampus. **(D)** Quantification of c-Fos cell count showed a significant (*p* < 0.05) increase in c-Fos staining in the 1 month old A30P mice. **(E)** Comparing 1 and 2–4 months of age in WT revealed no significant differences (*p* > 0.05), but in A30P mice **(F)** there was a significant decrease from 1 to 2–4 months of age suggesting a biphasic change in c-Fos expression in A30P mice. *p < 0.05, **p < 0.01.

Early gene transcription factors such as c-Fos and ARC are up-regulated in response to acute increases in neuronal activity (Kawashima et al., 2014), including during seizures (Willoughby et al., 1997). However, down-regulation of c-Fos in the hippocampus has been reported following presumed, chronic network hyperactivity in transgenic murine models of PD/DLB (Morris et al., 2015; Singh et al., 2019). As an indirect marker of neuronal activity, we conducted IF staining, initially in the 2–4 months cohort and found c-Fos+ cell nuclei scattered throughout the hippocampus in both WT (Figure 2Ai) and A30P mice (Figure 2Aii). As we have previously recorded interictal activity *in vitro* in CA3 of the A30P mice at this age (Tweedy et al., 2021), we quantified c-Fos+ cells in this region and found a significantly lower count at 2–4 months in A30P male mice compared to age matched control mice (Figure 2B). In A30P mice the median count of c-Fos+ nuclei were 35.3/mm^2^, (IQR 19.33–52.59; *n*/*N* = 23 sections/six mice) vs. a WT median count of 67.22/mm^2^ (IQR 43.22–75.53; *n*/*N* = 23 sections/six mice; *p* < 0.05, Mann Whitney test).

Because abnormal mutant hα-syn expression is already present in A30P mice at 1 month of age (Figure 1), it is possible that by 2–4 months of age network activity levels could have already been elevated for several weeks, resulting in this down-regulation of c-Fos (Morris et al., 2015; Singh et al., 2019). To test this hypothesis, we also measured c-Fos in sections from mice aged 1 month. Interestingly, the 1-month-old cohort of A30P mice showed an up-regulation of c-Fos expression in CA3. WT c-Fos expression at 1 month was similar to that seen at 2–4 months (Figure 2Ci), however, we found a significantly higher number of c-Fos+ cell nuclei in A30P mice (Figure 2Cii), consistent with a more acute hyperexcitable state. In A30P mice the median c-Fos+ nuclei count was 197.2 per mm^2^ (IQR 148.6–220; *n*/*N* = 8 sections/four mice) vs. WT median count 57.9 per mm^2^ (IQR 26.11–95.75; *n*/*N* = 8 sections/four mice; ^*^p < 0.05, Mann Whitney test). Comparing the c-Fos+ count between 1 and 2–4 months in WT mice (Figure 2E) showed no significant change. However, there was a statistically significant decrease in c-Fos+ count in the A30P mice (Figure 2F) between 1 and 2–4 months (*p* < 0.01). Overall, the biphasic profile of c-Fos expression suggested an initial increase in neuronal activity resulting in increased c-Fos expression in the A30P hippocampus at 1 month of age, but a presumed persistent hyperactivity, leading to down regulation of c-Fos gene expression by 2–4 months of age.

There is considerable evidence suggesting a link between hyperexcitability and neuroinflammation (Vezzani and Viviani, 2015; Li et al., 2023), thus we used IF staining to quantify changes in astrocytes and microglia in the A30P mice at both 1 month and 2–4 months of age.

### Increase in reactive astrocytes in the hippocampus in 2–4 months old A30P mice

**Figure 3:**
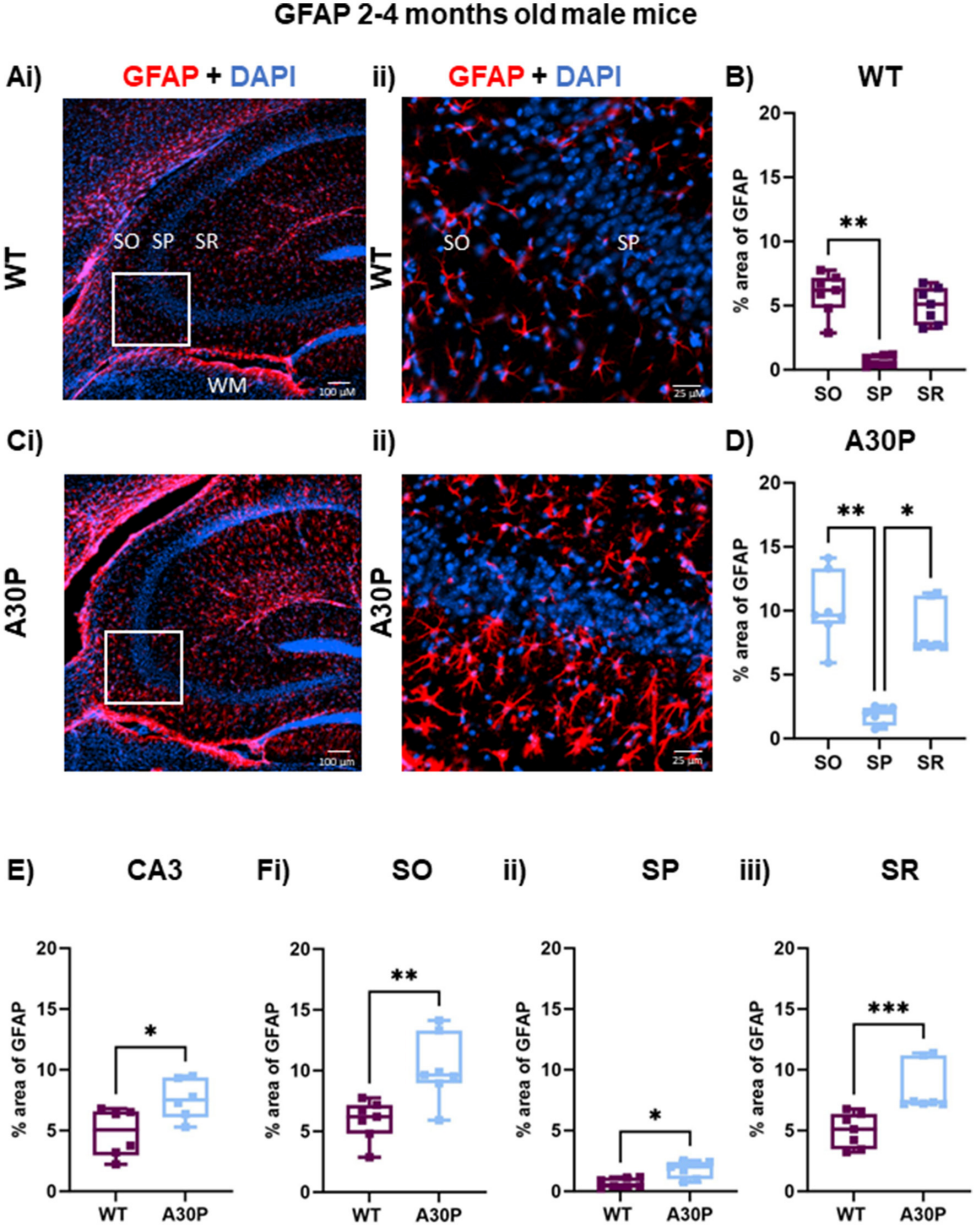
Increased reactive astrocytes in 2–4 months old male A30P mice. **(Ai–ii)** IF staining for GFAP (red) with DAPI counterstain (blue) in male WT 2–4 months old mice shows several GFAP+ astrocytes in hippocampus, especially in the white matter (WM). **(B)** Quantification of % area of GFAP across the *stratum oriens* (SO), *stratum pyramidale* (SP) and *stratum lucidum/radiatum* (SR) showed astrocytes were largely absent from the pyramidal cell layers. **(B)** There was a significant difference in % area GFAP between SO and SP in the WT mice (*p* < 0.01, Friedman's test). **(Ci–ii)** In the A30P mice there was a marked increase in % area GFAP across the whole of the hippocampus and the expanded view again showed fewer GFAP+ cells in the SP. **(D)** There was a significant difference in % area GFAP between SO and SP (*p* < 0.01) and SR and SP (*p* < 0.05) in A30P mice. **(E)** Overall, there was a significant increase (*p* < 0.05) in % area GFAP in A30P mice and this also seen when comparing each in each layer between WT and A30P mice **(Fi–iii)**, with the largest change in % area GFAP in the A30P mice observed in SO. *p < 0.05, **p < 0.01, *** *p* < 0.001.

The c-Fos experiments outlined above suggested chronic hippocampal excitability in male A30P mice at 2–4 months of age, so we first conducted IF studies of astrocytes in male WT and A30P mice at that age. Reactive astrocytes were immunostained using an antibody to GFAP (Figure 3) and visualization of the GFAP staining in male WT mice aged 2–4 months (Figures 3A, B) suggested a distinct laminar distribution of reactive astrocytes across the CA3 layers with few astrocytes present in *stratum pyramidale* (SP). Analysis of the laminar distribution in WT mice (Figure 3B) showed a significant difference between layers SO and SP (*p* < 0.01, *n*/*N* = 37 sections/six mice; Friedman's test followed by Dunn's multiple comparison). In male A30P mice there was clearly more GFAP expression (Figures 3Ci–ii), but the laminar distribution again showed significant differences (Figure 3D) between layers SO and SP (*p* < 0.01) and between SR and SP (*p* < 0.05), with again fewer astrocytes in SP (*n*/*N* = 46 sections/seven mice; Friedman's test followed by Dunn's multiple comparison).

Overall, there was a notable astrocytosis evident in male A30P mice with a significant increase (% area) of GFAP immunoreactivity (Figure 3E) compared to age-matched WT mice (WT median area 5.0, IQR 2.9–6.5%; *n*/*N* = 37 sections/six WT mice; A30P median area 7.5, IQR 6.0–9.3%; *n*/*N* = 46 sections/six mice; *p* < 0.05, Mann Whitney test). In view of the laminar differences noted above we also compared % area GFAP in WT and A30P in each layer and found significant differences in all three layers of the CA3 region (SO *p* < 0.01, SP *p* < 0.05, SR *p* < 0.001, Mann Whitney tests).

**Figure 4:**
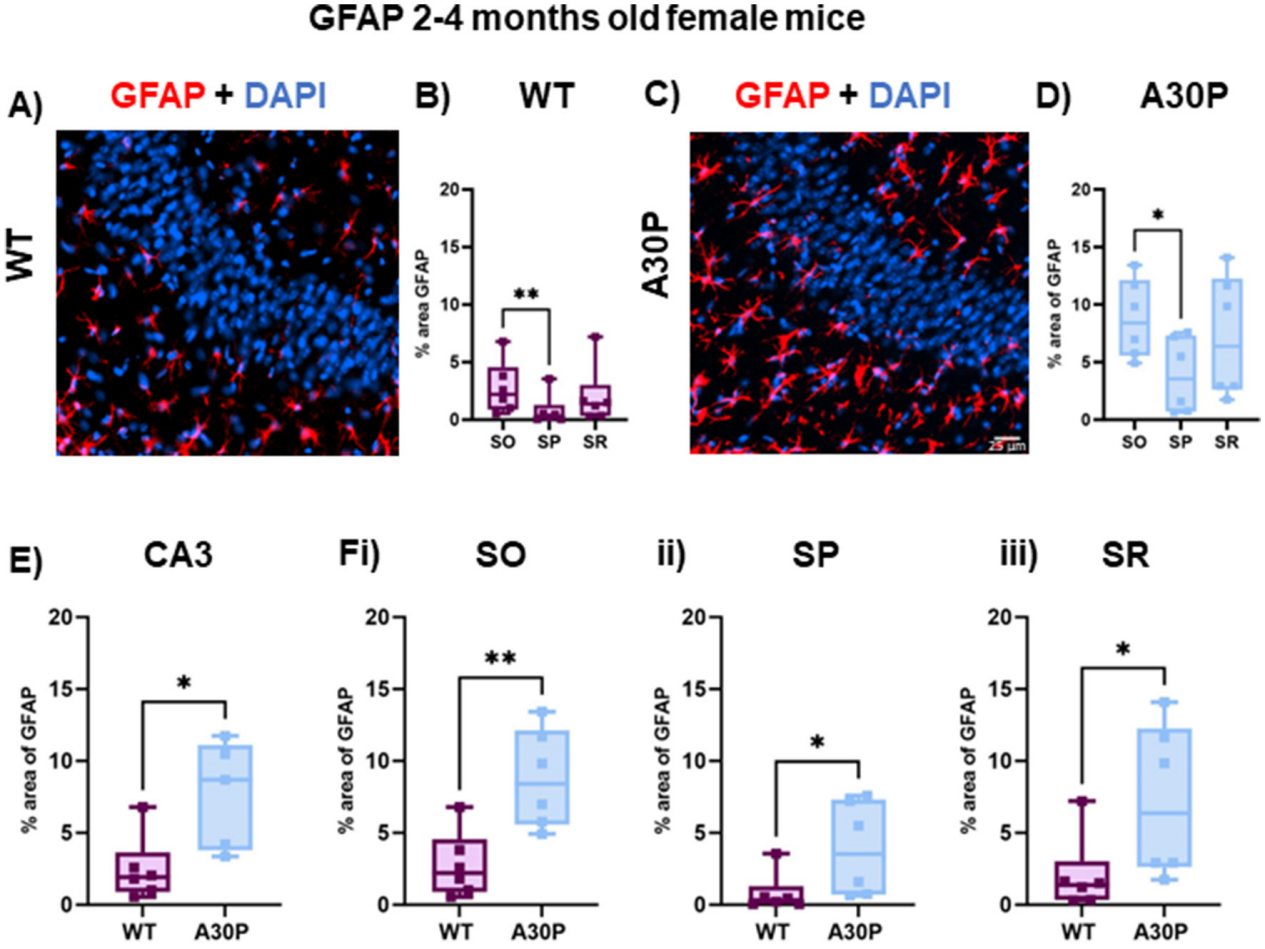
Increased reactive astrocytes in female 2–4 months old A30P mice. **(A)** IF staining for GFAP (red) with DAPI counterstain (blue) in female WT 2–4 months old mice. **(B)** Quantification of % area of GFAP across the *stratum oriens* (SO), *stratum pyramidale* (SP) and *stratum lucidum/radiatum* (SR) showed astrocytes were largely absent from the pyramidal cell layers. There was a significant difference in % area GFAP between SO and SP in the WT mice (*p* < 0.01, Friedman's test). **(C)** IF staining for GFAP (red) with DAPI counterstain (blue) in female A30P 2–4 months old mice. **(D)** There was a significant difference in % area GFAP between SO and SP (*p* < 0.05, Friedman's test). **(E)** Overall, there was a significant increase (*p* < 0.05) in % area GFAP in female A30P mice and this also seen when comparing each in each layer between WT and A30P mice **(Fi–iii)**, with the largest change in % area GFAP in the A30P mice observed in SO. *p < 0.05, **p < 0.01.

Because there are known sex-dependent differences in glial cells and neuroinflammatory responses in human disease and rodent models of neurodegeneration (Chowen and Garcia-Segura, 2021; Bourque et al., 2023), we also quantified GFAP expression levels in female WT and A30P mice at 2–4 months of age (Figure 4). Similar results were found in female mice as there was also a laminar distribution of the GFAP+ cells in both WT (Figures 4A, B) and A30P (Figures 4C, D) female mice, with fewer GFAP+ astrocytes located in the pyramidal cell layer. Furthermore, analysis of the laminar distribution in WT (Figure 4B) and A30P (Figure 4D) female mice showed a significant difference between SO and SP in both WT (*p* < 0.01) and A30P (*p* < 0.05) (Friedman's test followed by Dunn's multiple comparison). There was also a significant increase in % area GFAP+ astrocytes (Figure 4E) across the whole of the CA3 in female A30P mice (WT median area 1.9, IQR 0.89–3.6%, *n*/*N* = 26 sections/six mice; A30P median area 8.6, IQR 3.7–11.1%, *n*/*N* = 26 sections/six mice; *p* < 0.05, Mann Whitney test). Again, in view of the laminar differences noted in the distribution of astrocytes we also compared % area GFAP in female WT and A30P in each layer and found significant differences in all three layers of the CA3 region (SO *p* < 0.05, SP *p* < 0.01, SR *p* < 0.05, Mann Whitney tests). Our data, therefore, demonstrated that in both male and female mice, the expression of human mutant α-syn evoked a significant increase in reactive GFAP+ astrocytes in the CA3 region.

In view of the age-dependent changes seen in the c-Fos expression between the 1 month and 2–4 months old cohorts of A30P mice outlined above (Figure 2) we also assessed GFAP expression levels in a small cohort of 1 month old male mice. However, although there was a trend for an increase in % area of GFAP immunoreactivity there were no significant differences in % area of GFAP either across the whole of the CA3 region (WT median area 3.7, IQR 3.4–4.1%, *n*/*N* = 12 sections/three mice; A30P median area 6.7, IQR 6.0–8.0%, *n*/*N* = 12 sections/three mice; *p* > 0.05, Mann Whitney test), or across the different layers (SO, SP, SR *p* > 0.05, Mann Whitney tests, data not shown).

### Increase in reactive microglia in the hippocampus in both 1 month and 2–4 months old A30P mice

**Figure 5:**
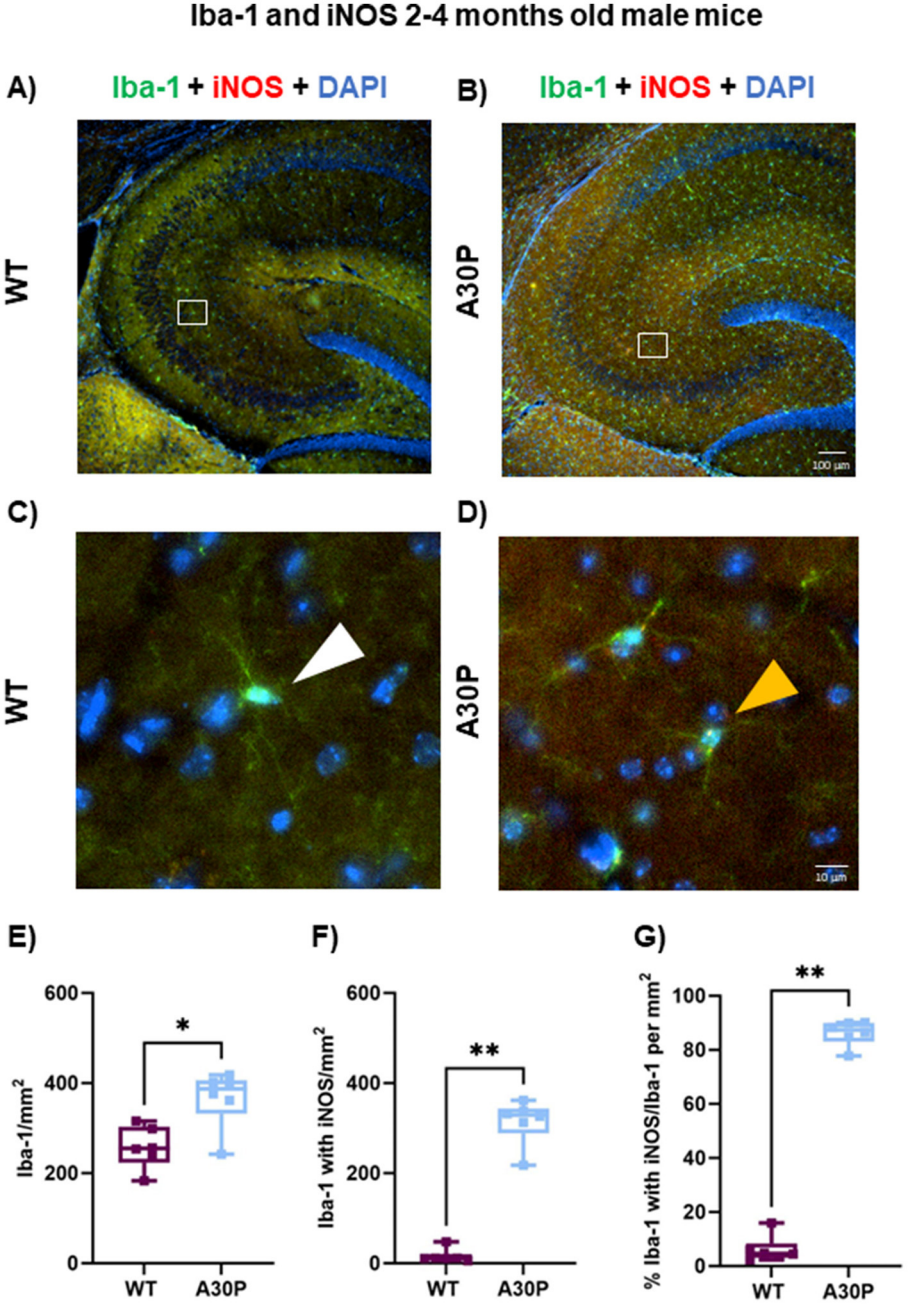
Marked increase in Iba-1+ cells expressing iNOS in male A30P mice. IF staining of Iba-1 (green) and iNOS (red) with DAPI counterstain (blue) in 2–4 months old male **(A)** WT and **(B)** A30P mice. Microglia are seen in both genotypes across the hippocampus but there was a clear increase in Iba-1+ cells in the A30P mice. No laminar differences were noted for Iba-1+ which were present in all layers. In the expanded view **(C)** for WT no iNOS co-labeling with Iba-1 is evident (white arrows), but Iba-1+ cells in the A30P mice **(D)** show clear iNOS staining (yellow arrows). **(E)** Quantification of the total number of Iba-1+ cells showed a significant increase (*p* < 0.05) in A30P mice compared to WT. **(F)** Significantly more Iba-1+ cells co-labeled with iNOS in A30P mice (*p* < 0.01). **(G)** While the number of Iba-1+ cells with iNOS divided by the total number of Iba-1+ cells for both WT and A30P groups showed that nearly all Iba-1+ cells were also iNOS positive in the A30P mice (*p* < 0.01). *p < 0.05, **p < 0.01.

Microglia can be immunostained for Iba-1 although this marker is not specific for reactive microglia, therefore, we co-stained Iba-1+ cells for iNOS, which is expressed by reactive microglia as well as some neurons (Galea et al., 1992). In contrast to the laminar distributions seen with the GFAP expression in astrocytes (Figures 3, 4), the microglia appeared equally distributed across all hippocampal layers in both the male 2–4 months old WT and A30P mice (Figures 5A, B). Few microglia in WT mice were co-labeled with iNOS (Figure 5C, white arrow), but in the A30P mice many Iba-1+ cells also expressed iNOS (Figure 5D, yellow arrow).

To quantify the number of reactive microglia we counted Iba-1+ cells in total (Figure 5E), and then Iba1+ cell with iNOS (Figure 5F). There was a statistically significant increase in the total number of Iba-1+ cells in the male A30P mice compared to WT (WT median count 255 per mm^2^, IQR 223–303, *n* = 12 sections/six mice, vs. A30P median count 387 per mm^2^, IQR 332–406, *n*/*N* = 13 sections/six mice, *p* < 0.05, Mann Whitney test). We also found a highly significant increase in the number of Iba-1+ cells that co-labeled with iNOS (Figure 5F) in the 2–4 months male A30P mice (WT median count 11 per mm^2^, IQR 9.3–20.45, *n*/*N* = 12 sections/six mice, vs. A30P median count 329, IQR 289–343 *n*/*N* = 13 sections/six mice, *p* < 0.01, Mann Whitney test). Overall, the proportion of Iba-1+ cells co-labeled with iNOS was significantly greater (*p* < 0.01) in A30P mice (87% of the total Iba1+ cells in A30P sections), compared to WT mice (4.6% of the total Iba-1+ cells in WT sections, Figure 5G). These data demonstrated a marked increase in the number of reactive microglia in the male 2–4 months old A30P mice compared to WT.

**Figure 6:**
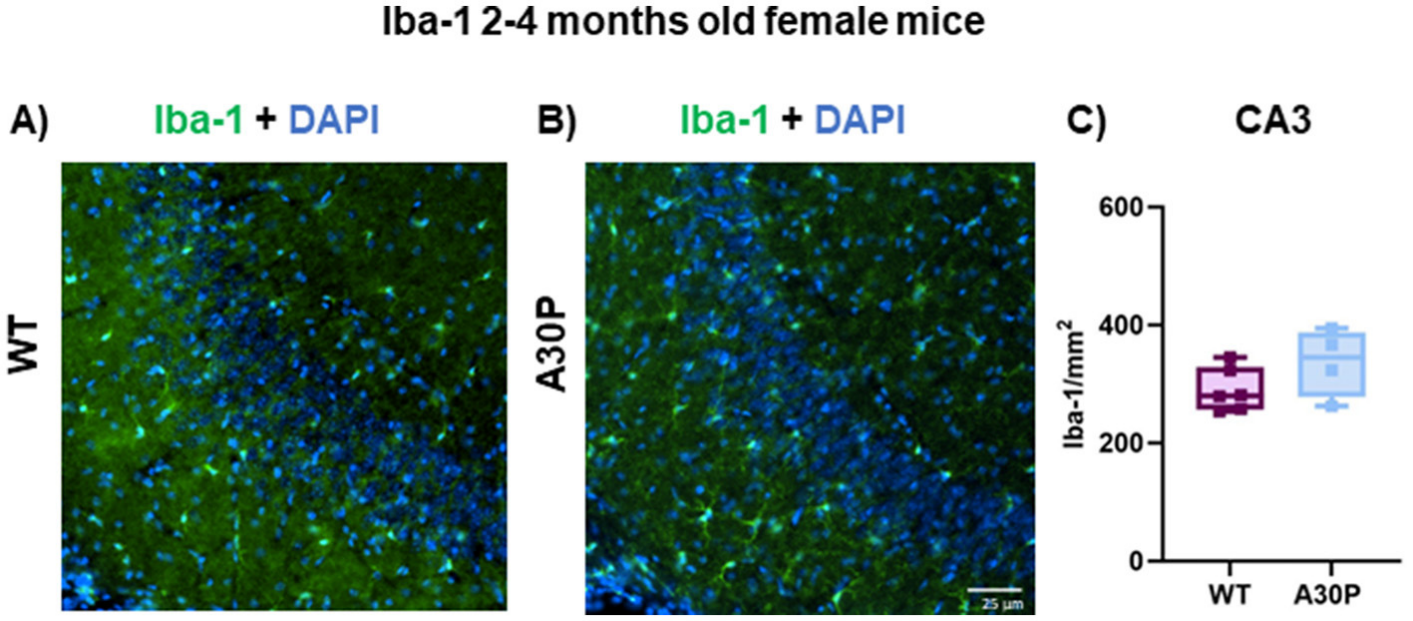
Iba-1 expression in female 2–4 months old A30P mice. IF staining of Iba-1 (green) and DAPI (blue) in 2–4 months old female **(A)** WT and **(B)** A30P mice. **(C)** Quantification of the total number of Iba-1+ cells showed no significant difference (*p* > 0.05) in A30P mice compared to WT.

Interesting molecular and functional sex-related differences in microglia have been reported in mice (Guneykaya et al., 2018; Villa et al., 2018) thus we also quantified Iba-1 expression levels in female WT and A30P mice at 2–4 months of age (Figures 6A, B). For female mice there was a slight increase in Iba-1 count but this did not reach statistical significance (Figure 6C). The total number of Iba-1+ cells was (WT median count 280 per mm^2^, IQR 257–329, *n* = 18 sections/six mice, vs. A30P median count 345 per mm^2^, IQR 278–387, *n*/*N* = 12 sections/four mice, *p* < 0.05, Mann Whitney test).

**Figure 7:**
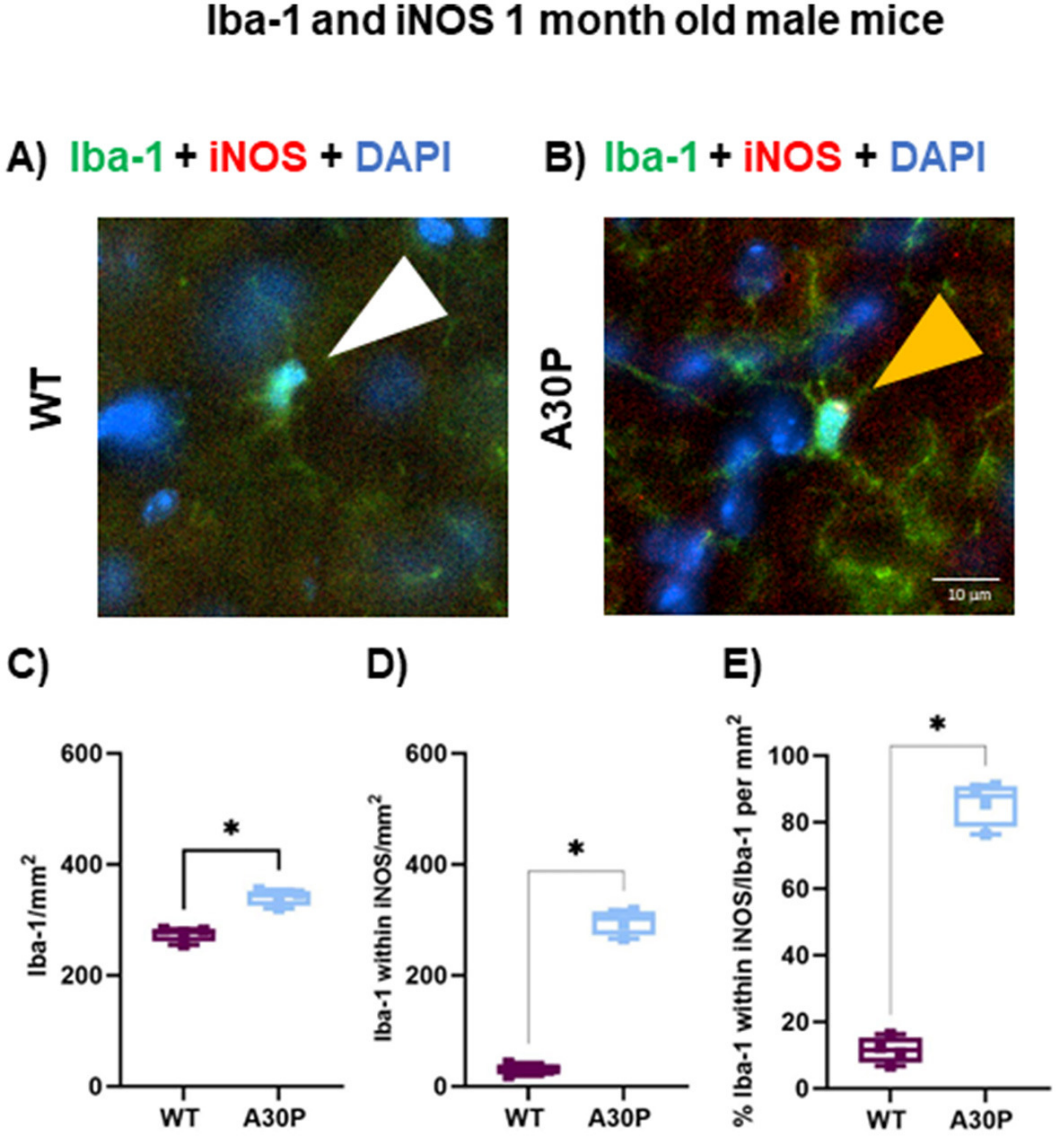
Iba-1+ cells expressing iNOS in and 1-month mice. IF in **(A)** WT and **(B)** A30P 1 month old male mice. **(C)** Quantification of the density Iba-1+ cells showed a significant increase (*p* < 0.05) in A30P mice compared to WT. **(D)** Counting the number of Iba-1+ cells that co-labeled with iNOS showed a significant increase in A30P mice (*p* < 0.05). **(E)** The proportion of Iba-1+ cells also expressing iNOS was significantly higher A30P mice than in WT (*p* < 0.05). *p < 0.05.

Lastly, we compared the number of Iba-1+ cells co-stained with iNOS in the CA3 region of the hippocampus in a small (*N* = 4) cohort of 1 month old A30P mice (Figures 7A, B). In contrast to the GFAP results outlined above, with Iba-1 a significant increase in reactive microglia was identified even at this very juvenile age. There was a statistically significant increase in the total number of Iba-1+ cells in 1 month old A30P mice (Figure 7C) compared to WT (WT median count 279 per mm^3^, IQR 262–283, *n*/*N* = 11 sections/four mice, vs. A30P median count 342 per mm^3^, IQR 326–352, *n*/*N* = 14 sections/four mice, *p* < 0.05, Mann Whitney test). We also found a significant increase in the number of Iba-1+ cells that co-stained with iNOS (Figure 7D) in the 1 month old A30P mice (WT median count 32 per mm^2^, IQR 21.8–39.8, *n*/*N* = 11 sections/four mice, vs. A30P median count 302 per mm^2^, IQR 273–315 *n*/*N* = 14 sections/four mice, *p* < 0.05, Mann Whitney test). Overall, the proportion of Iba-1+ cells co-stained with iNOS was significantly greater (*p* < 0.05) in A30P mice (88% of the total Iba-1+ cells in A30P sections) (Figure 7E), compared to WT mice (11.5% of the total Iba-1+ cells in WT sections). These data demonstrated a marked increase in the number of reactive microglia in the 1 month old A30P mice compared to WT.

## Discussion

Our data demonstrated interesting age-dependent changes in c-Fos expression as a marker of acute and chronic neuronal activity. In addition, we found a notable early neuroinflammatory response to the expression of human mutant α-syn in the A30P mice which was associated with an increase in reactive astrocytes and microglia compared to age matched control mice as indicated by greater expression of GFAP and iNOS immunoreactivity, respectively. The changes in microglia were seen in A30P mice as early as 1 month of age, and at 2–4 months of age there was an increase in both reactive astrocytes and microglia.

### Distribution of α-syn to deep pyramidal layer cells

We noted that hα-syn immunostaining in the A30P mice was particularly strong in the deep pyramidal cell layer in CA3 of the hippocampus (Slomianka et al., 2011) in both juvenile mice (1 month old) and adult mice (2–4 months old). This preferential distribution to deep SP is interesting as it is now clear that hippocampal pyramidal cells are less homogenous than previously thought, with molecular, morphological, and functional heterogeneity seen in the proximo-distal location of cells in CA3 (Sun et al., 2017) and CA1 (Valero and de la Prida, 2018) regions. Thy-1 is an immunoglobulin family member thought to be expressed in excitatory projection neurons, although Proskurina and Zaitsev (2021) found fast-spiking inhibitory interneurons also expressed channelrhodopsin-2 when under a Thy-1 promoter. Several studies using the Thy-1 promoter to express different transgenes have shown expression of the target gene in only a subpopulation of hippocampal neurons (Feng et al., 2000; Dobbins et al., 2018; Proskurina and Zaitsev, 2021). Interestingly, Dobbins et al. (2018) found expression of channelrhodopsin under Thy1 favored deep layer CA1 pyramidal cells, and recent studies in the transgenic THY-Tau22 model of tauopathy, where tau is under the control of the Thy1.2 promoter, reported preferential of expression of tau in the deep layers of CA1 pyramidal cells (Viney et al., 2022). These data are, therefore, consistent with our observations that hα-syn expression in the A30P was largely in the deep CA3 pyramidal cell layer.

### Down-regulation of c-Fos in A30P mice may reflect chronic hyperexcitability

The immediate early gene c-Fos is often used as an indirect marker of neuronal activity (Kawashima et al., 2014). We found an interesting biphasic pattern of c-Fos expression with higher expression in young (1 month old) A30P mice compared to WT, which was followed by a reduction in c-Fos at 2–4 months of age to below that of the age matched control mice. Reduced c-Fos expression has been observed in other α-syn transgenic lines including mice expressing the A53T mutation (Singh et al., 2019), and wild type hα-syn mice (Morris et al., 2015; Torres et al., 2021). In these studies, reduced c-Fos was thought be associated with hippocampal remodeling because of chronic hyperactivity. As far as we are aware this is the first study in α-syn transgenic mice showing that c-Fos expression changes are biphasic, with an initial increase in c-Fos in juvenile mice, likely reflecting an early increase in neuronal excitability, followed by the later down-regulation following chronic network excitability changes.

c-Fos positive nuclei are seen in pyramidal cells but also in most interneuron subtypes, including PV+ cells, but not calretinin positive interneurons (Staiger et al., 2002). However, although widely discussed as being a marker of neuronal activity there has long been evidence suggesting that at least some glial cells also express c-Fos. Evidence suggests c-Fos is up-regulated in reactive astrocytes and microglia [reviewed in detail in Cruz-Mendoza et al. (2022)]. A qualitative assessment of the location of c-Fos positive nuclei in our study showed they were located across all layers of the CA3 region, thus are not specifically confined to the cells in the pyramidal cell layer. Torres et al. (2021), using the A53T transgenic mouse line, found some PV+ cells were also c-Fos positive. In our study we have not co-labeled c-Fos with specific cell markers, but determining whether pyramidal cells, PV+ cells, or glia cells show changes in c-Fos expression will be interesting next steps.

Our data demonstrated a down regulation of c-Fos at 2–4 months in A30P mice as seen in other α-syn transgenic lines that exhibited chronic hyperexcitability (Morris et al., 2015; Singh et al., 2019) and is consistent with our previous findings of network interictal discharges in mice of the same age (Tweedy et al., 2021). Combined these data suggest chronic hyperexcitability is occurring at the earliest stages of disease progression in the A30P mice. Understanding the causes of network excitability are important because AD patients with seizures showed an earlier onset of symptoms by 5.5 years compared to non-epileptic AD patients (Vossel et al., 2016). Patients with DLB exhibit visual hallucinations and cognitive fluctuations (McKeith et al., 2020), symptoms which may reflect changes in cortical network excitability (Taylor et al., 2016). More recently subclinical epileptiform activity, in the form of interictal discharges, were observed using ear-EEG in DLB patients with twice the prevalence seen in control subjects (Musaeus et al., 2023).

### The role of neuroinflammation in early disease stages

Astrocytes play an important role in the clearance of protein aggregates including α-syn (Giusti et al., 2024), which can involve the transfer of α-syn from neuron to astrocytes (Lee et al., 2010). We found a significant increase in reactive astrocytes number and/or sizes as indicated by an increase in % area GFAP immunoreactivity in both male and female A30P mice compared to WT, even at the pre-symptomatic stages at 2–4 months of age. We also found an interesting laminar distribution of GFAP+ astrocytes which were found to be largely absent from the pyramidal cell layer. In the very young juvenile animals aged just 1 month, however, the increase in the % area of GFAP did not quite reach statistical significance. Although a small sample size, the data from 1 month of age suggested that at the very youngest age tested in this study, changes in astrocytes may be less marked.

Along with their homeostatic functions astrocytes regulate inhibitory neurotransmission through GABA uptake and release (Kilb and Kirischuk, 2022; Andersen et al., 2023), and thus play an important role in regulating network excitability. Changes in the regulation of GABA synthesis or release because of altered astrocyte function (Liddelow and Barres, 2017) could, therefore, contribute to the hyperexcitable hippocampal network state seen in both the A30P mice (Tweedy et al., 2021), and other transgenic hα-syn lines (Morris et al., 2015; Peters et al., 2020). Consistent with this, recent studies have suggested a role for astrocytes in the generation of seizure activity (Chan et al., 2019).

Along with the increase in reactive astrocytes in A30P mice observed in this study we also found changes in microglia. Recently microglia have been shown to remove and transfer aggregated α-syn load via gap junctions or tunneling nanotubes (Scheiblich et al., 2021). α-syn also triggers microglial activation via the Toll-like receptors (TLR2 and TLR4) leading to release of downstream pro-inflammatory mediators (Fellner et al., 2013). Unlike the distribution of astrocytes which predominated in *stratum oriens* and *stratum radiatum* we found that the microglia cells appeared evenly distributed across all layers of the hippocampus as previously reported (Jinno et al., 2007). The total count of Iba-1+ cells was significantly increased only in the male A30P mice. Whether this reflects a reduced neuroinflammatory response in female A30P mice needs to be explored in further studies. In the male mice co-staining of Iba-1+ microglia cells with iNOS showed a significant increase in the proportion of Iba-1+ cells that co-expressed iNOS in the male A30P mice at both 1 and 2–4 months of age compared to wild type mice. In fact, the majority of Iba-1+ cells (87%) in the 2–4 months old male A30P mice also expressed iNOS, compared to only a small proportion in wild type mice (4.5%), suggesting that most microglia had adopted a reactive disease-related phenotype.

The use of “reactive” or “resting” terminology has recently been challenged as microglia are never actually resting, but are always active and “surveying”, and they exhibit a wide range of disease and age-related morphological changes (Paolicelli et al., 2022). We have not conducted detailed morphological analysis of the shape changes of microglia (or astrocytes) in this study, but this would be important explore in the future. Interestingly microglial activation was shown to peak prior to activation of astrocytes (Bantle et al., 2021) which may explain the increase in Iba-1+ cells expressing iNOS, but no change in GFAP, in 1 month old male A30P mice. Previous studies using different models of hα-syn expressing mice have reported evidence of increased glial reactivity (Gomez-Isla et al., 2003; Watson et al., 2012; Marxreiter et al., 2013; Allen Reish and Standaert, 2015; Tanriover et al., 2020; Gentzel et al., 2021; Torres et al., 2021; Rauschenberger et al., 2022). However, there is considerable variation across these studies in the models used, brain regions explored, and the age of the mice investigated.

Microglia act as the first line of defense and are also associated with the production of pro-inflammatory cytokines including tumor necrosis factor alpha (TNFα) and different interleukins IL-1β, IL-6 and IL-12. These cytokines increase neuronal excitability (Vezzani and Viviani, 2015) and thus chronic neuroinflammation could contribute to the network hyperexcitability seen in neurodegenerative disease. We have shown early indirect changes in neuronal excitability, as suggested by the changes in c-Fos expression, and a progressive increase in neuroinflammation from 1 to 2–4 months of age. Recent significant studies have shown that microglia play a complex role in the control of neuronal excitability by sensing ATP release arising from neuronal activity, which then activates a suppressive feedback pathway via adenosine (Badimon et al., 2020). In addition, microglia have been shown to respond to excitatory glutamate driven neuronal activity with increased process extension and retraction and decreased motility which can lead to increased network hyperexcitability (Merlini et al., 2021). These, and other studies, suggest microglia are significant regulators or network excitability which will have important implications for elucidating the mechanisms that link network hyperexcitability, neuroinflammation and neurodegeneration. Understanding neuroinflammatory changes linked to α-syn is particularly important because PD patients with a pro-inflammatory profile exhibit a faster decline (Kouli et al., 2020).

The incidence of DLB is greater in men than in women but pathology and clinical features vary with gender (Oltra et al., 2024). One recent study using a mouse model of PD showed that α-syn accumulation was age-dependent, but with male mice being more severely affected by α-syn toxicity than females (Lamontagne-Proulx et al., 2023). Microglia from female mice have also been shown to be neuroprotective (Villa et al., 2018), a function which is preserved when transferred into the brains of male mice. Overall, there is considerable evidence now that sex/gender differences in glial cells contribute to the pathophysiology of neurodegenerative diseases.

## Conclusions and future directions

Overall, the results of this study demonstrated that marked gliosis is occurring long before the onset of any cognitive or motor deficits which occur at 12 and 14+ months respectively in the A30P mouse line (Kahle et al., 2000; Freichel et al., 2007). In addition, we have shown a close association between early neuronal hyperexcitability and neuroinflammation. Chronic hyperexcitability, which we have previously observed (Tweedy et al., 2021), was proposed to account for the downregulation of c-Fos seen in A30P mice aged 2–4 months. Changes in c-Fos were coupled to a marked increase in both reactive astrocytes and microglia. Evidence from human studies in patients with DLB also suggests that neuroinflammatory changes occur early (Loveland et al., 2023). In contrast, in later disease stages abnormal dystrophic microglia and astrocytes are observed, and there is a reduced neuroinflammatory response, possibly due to immune senescence (Bachstetter et al., 2017). Therefore, assessing whether neuroinflammation declines with disease progression in the A30P mice will be important.

## Data availability statement

The raw data supporting the conclusions of this article will be made available by the authors, without undue reservation.

## Ethics statement

The animal study was approved by Home Office License and Newcastle University Ethics. The study was conducted in accordance with the local legislation and institutional requirements.

## Author contributions

IA-M: Writing – review & editing, Formal analysis, Investigation, Methodology. BD: Methodology, Writing – review & editing, Software. GC: Methodology, Writing – review & editing, Project administration, Supervision. FL: Project administration, Supervision, Writing – review & editing, Conceptualization, Resources, Writing – original draft.
